# Some Points in the Etiology, Pathology and Indicated Treatment of Diphtheria1Read before the Central New York Medical Association, Buffalo, N. Y., October 16, 1894.

**Published:** 1894-12

**Authors:** A. A. Young

**Affiliations:** Newark, N. Y.


### SOME POINTS IN THE ETIOLOGY, PATHOLOGY AND INDICATED TREATMENT OF DIPHTHERIA.[1]

By A. A. YOUNG, M. D., Newark, N. Y.

WELL do I remember the first case of diphtheria known to me. I was the victim, and my experience then gave me the impression that it was something more than a mere throat affection; an impression that has grown stronger even to the present hour. A few years later, the disease appeared in its most virulent form; deaths were then common; the skill of the physician was taxed to the utmost and that skill dependent upon occular perceptions and not upon thought induced by investigations; the physician could not say "I know, for I have mastered," but rather, I act as I have been told to act. As the membrane was the first indication of the approach of the disease, a diagnostic sign of it, diphtheria came to be regarded as a local affection and the throat the objective point of the physician's attack; the membrane must be removed, though he knew but little of its true character except it was poisonous. At the onset of this disease, or, indeed, any throat difficulty, the first service of the family physician was to cauterize the throat with a strong solution of nitrate of silver, or the solid stick even. It would have been as unfortunate for the physician to appear without his caustic as for the physician of earlier times to appear without his lancet, calomel or jalap. The treatment did not stop with the cauterization of the diphtheritic throat; preventive measures must be instituted. Johnnie and Joe, Susie and Annie must in turn submit to the process of cauterization and submit to the same solution and the same old swab just used upon the diphtheritic patient, and, upon the score of economy, sometimes the self-same old swab was preserved till next application. We smile at the acts of our predecessors, although they did the best they could; but, somehow, I fancy that twenty-five years hence the boys of the profession will smile over our oddities and inconsistencies as we smile today over those of our predecessors.

Concerning diphtheria there are still many mooted points, although a few seem to have been definitely settled, and among that number we may mention the following: First, that it is a highly contagious disease. Second, that it has a period of incubation. Third, that it runs a comparatively definite course, partaking of the nature of a self-limited disease. Fourth, that it is pro-

1. Read before the Central New York Medical Association, Buffalo, N. Y., October 16, 1894.

duced by a peculiar specific germ, the favorite seat of which, when fully developed, is in the fauces.

The question, and a most vital one, still remains debatable—namely, Is diphtheria a local or systematic affection? Are the systematic disturbances dependent entirely upon the presence of diphtheritic membrane, toxalbumen, or the presence of the specific germs within the blood or body? This question is vital, for correct therapeutics depend upon a correct answer.

Our first intimation or positive sign of the disease is the appearance of the membrane usually attached to the tonsils, palate or uvula, greyish-white in color, tough and adherent and apparently beneath the epithelium, through which it breaks a little later. The pseudo-membrane is found to be composed of necrosed epithelium, disintegrated leucocytes, pus, fibrin and bacilli, the latter scattered freely between the meshes. In conjunction with this membrane there is a swelling of the cervical glands and of the sub-maxillary glands, the latter being an important factor in differential diagnosis; the swelling of these glands appears as early as the membrane, and it is probable that their enlargement appears prior to membrane formation.

It is doubtless true that diphtheria consists of the deposition and development of a specific germ which may be deposited, and usually is, within the fauces; but it is my belief, a belief founded upon observation, that the specific germ is not developed at the point of deposition, that it is not congenial soil for its propagation, that it is developed within the system producing general malaise, which is usually well marked before the appearance of the membrane. From the study I have made I am fully convinced that the Imago, as found in the membrane, is developed from an ovum and that this ovum must be within the system during its development. If this be true, then, as well think of excising the diphtheritic membrane as resorting to local treatment to cure the disease, as to excise, by cautery, or otherwise, a primary syphilitic chancre and call this procedure a cure. Syphilis we know and acknowledge becomes constitutional at once from the time of contact, though many days do elapse before the presence of the disease is detected in other ways.

A further proof of the systemic character of diphtheria is shown from the fact that death occurs almost invariably from systemic poisoning and not from asphyxia, as is seen in membranous croup; what is apparently asphyxia usually consists of collapse of lung tissue, or paralysis of muscles, of respiration or circulation. That paralysis is oftentimes a sequela of diphtheria is not a theory but a fact, a paralysis that ofttimes remains after all other signs and symptoms of the disease have subsided. Pure mechanical obstruction of the respiratory tract by pseudo-membrane I have never seen, and I question if this condition should ever be taken into account as a *mode* of death, and this leads directly to the question, Is tracheotomy ever justifiable in diphtheria, and if so, why and when? You who approve it may answer.

The stomach as well as other abdominal organs suffer, and especially the former. Here we have inactivity of the peptonic glands and diminution of hydrochloric acid and sodium chlorides, due to the poisonous exudate coming into contact with the sensative membrane, producing partial inactivity of it. As a factor in producing this inactivity the general systemic poisoning must also be taken into account.

The urinary organs, either directly or indirectly, soon become involved; the urine is diminished in quantity, high colored and usually contains albumen, the presence of which means excessive uric acid and urea formations and their imperfect elimination; this abnormal condition increases with the severity of the disease and decreases as the disease progresses toward a favorable issue. In this regard there is a similarity between diphtheria and most of the acute febrile diseases; another indication of its systemic character.

Many more characteristics of this disease might be given, but it is needless, as you all are doubtless familiar with them.

It has been stated that diphtheria is apparently a self-limited disease, which is, as a rule, true; as it progresses the different phases which it presents are as constant as those of pneumonia or typhoid fever. Death may shorten this period, but if lengthened is due to infection at other foci at a later period than the primary lesion, if such the point of infection may be called. As a rule, the active stage of the disease extends over a period of ten days, after which comes a period of decline of some ten days more duration, when convalescence is fully established.

That diphtheria is a self-limited disease may be inferred by analogy. All of the infectious diseases run a more or less regular course; all are systemic affections having more or less marked local manifestations which sometimes predominate over the systemic ones.

All infectious diseases have their origin in the deposition and development within the body of germs peculiar to their kind, and these, too, have a favorite seat from which development proceeds, depending upon their character. The bacillus diphtheria chooses the fauces and adjacent parts; the pneumococcus the lung tissue; the bacillus typhosus Peyer's patches, and so on. These points of attack become later in each disease the nidus, the home of the fully developed germ. All life, as seen through a material organization, has a formative stage, markedly different from its perfect being as seen in its complete existence; so by analogy we say bacilli follow the same law; time is required for their full development and this time may represent the so-called period of incubation. It is during the development of the bacilli that we say the disease is progressive and when the end of their development is reached, that the disease has turned its course, that convalescence is established, although the danger line not passed. Nature now seeks to eliminate the poison, whether that poison be inanimate bacilli or toxalbumen, a substance produced by the growth of bacilli, and as toxalbumen, when present, renders germ life impossible.

The union of two or more substances, chemically or biologically, form by that union a substance unlike any of its component parts, and, in a large degree, such substance by its presence renders inoperative chemical or biological action. This seems to be a law of Nature, not in exact accordance with the law of *Similia Similibus Curantur*.

Diphtheria cannot be a local affection, for the lesion is not commensurate with the systemic disturbances from its onset to its close. That diphtheritic germs are found in the fauces and elsewhere, with no systemic disturbances, is a fact, but not proof that the possessor is ill with the disease; if diphtheria ever be local, it is now, just prior to the entrance of the germ through the membrane, prior to any and all systemic disturbances. The assertion that diphtheria can exist actively prior to systemic disturbances is open to ridicule and justly so; as well say the whole human family is continually ill because of constant contact with bacteria. In any infectious disease it is not till some germ or germs permeate the delicate tissues, forcing a way into the circulation, can germ disease be said to exist. Nature is practically unchangeable, and because of that fact, science is possible. The worm-like larva is no less a butterfly than the beautiful *imago*, but to develop it requires peculiar and well-known conditions. So is it with the bacillus diphtheria; when it finds its peculiar element, then and not till then will it develop and come forth as a perfect being, finding its final home in the diphtheritic membrane, of which it forms a part. Not till the germ begins its development in the developing media of the body, do the systemic disturbances begin, disturbances that mark the beginning and progress of the disease. A leopard cannot change his spots, neither can a germ in its process of development be local today and systemic tomorrow, for a transition from one condition to another requires a change in food, a change in growth, a change in its entire being. Nature does not work that way. She is systematic and consistent. She has, therefore, made rational treatment possible, though it is still in the future. That treatment may be and the indications are that it will consist of the introduction into the system of antitoxine, toxalbumen, or educated bacilli, the object of which will be to eliminate, destroy or render inert that peculiar element which the germs require for their growth, thus rendering development impossible. May the day speedily come when the disease shall be treated, whatever that treatment be, upon a rational, scientific basis and not by one dependent upon "signs and symptoms."

A word now in regard to treatment: not the treatment, but my treatment, for specific medication is as yet unknown. This is twofold: first, prophylactic; second, medicinal. Of the former, but little more can be said than complete isolation of the infected from the apparently non-infected, and for the latter a good nutritious diet, fresh air and sunshine, but otherwise a severe letting alone. Of the medicinal treatment, local applications form but a minor part. I seldom make an external application of poultices or similar appliances, for, if the theory be correct, what good can come from their use? They must interfere with respiration and thus indirectly affect circulation, which is already weak. Steam has been used after the line of demarkation became prominent between the pseudo- and true membrane, to hasten exfoliation and expulsion; the impregnation of steam with medicinal agents I regard as almost worthless. As a gargle and mouth wash, permanganate of potassium, two to four grains to eight ounces of water, is used frequently. Of the full germicidal properties of this solution I have some doubt, but I get a moral effect; it is a shield from unjust criticism. The family and friends imagine something is being done for the throat, for to them it is a true throat affection in spite of all the physician may say to the contrary.

Internally, bichloride of mercury is given, not alone for its germicidal effects, but to coagulate the albumen and thus carry off in a mechanical way some bacilli that may have been swallowed. Another benefit to be derived from the use of this drug is the increase of red corpuscles in the blood, over the production of which it exerts a favorable influence. Strychnine is given in varying doses, to counteract the tendency to paralysis, watching closely its physiological and toxicological effects.

Pilocarpin is prescribed in sufficient quantity to produce a moderate amount of perspiration, and at the same time the quantity of urine is also increased, thus favoring the proper elimination of uric acid and other urates. For this purpose, I regard this drug as par-excellence, since it produces the elimination of uric acid and urea by the skin as well as by the urinary organs. Since there is a loss of hydrochloric acid, this also is supplied together with some of the sodium compounds, sodium chloride being preferred.

Since diphtheritic poison interferes markedly with digestion, some of the so-called digestants are used, my preference now being for papoid, not to dissolve the membrane, for I regard this a questionable procedure, but to keep in solution the food products in the alimentary canal, which appears a necessity. Iron is seldom used, except during the period of convalescence, and then sparingly.

This line of treatment has given me good results—results that compare favorably with other practitioners—a line that I shall doubtless follow till something more is known of that dreaded disease, diphtheria.

DISCUSSION.

Dr. J. O. Roe: Mr. Chairman, I do not think anyone would question the fact of diphtheria being a constitutional disease, but the point was brought forward first by Œrtel that it originated locally, that it had a local origin, that it was a local affection and that the constitutional affection was purely secondary to the local affection. Now, I do not think the doctor's paper will controvert that fact, that the systemic affection does not spread from a local process; that the inoculation usually takes place in the throat, where is provided an inviting field, and from that the general affection takes place. The treatment, which the doctor, of course, did not have time to touch upon, will carry out this point. No one would confine himself to the treatment of local process. He would simply destroy this local process and prevent further infection and then present his main treatment to the treatment of the systemic trouble.

Dr. N. S. Jacobson: I do not wish, Mr. Chairman, to occupy too much time of the society, but I have a word to say upon this subject, and that is briefly this: If there is any one thing established today; if bacteriology has accomplished any one thing today, it is an assurance of the fact that diphtheria is always primarily local in its origin. It has shown that the germs are found in the throat. It has shown that they are not found at all in the blood. It has shown that the disease is started in the throat and that is absolutely primarily its origin. In the two minutes that are allowed, of course, this question cannot be entered into, but if anyone desires positive proof of this, let him read the report of Welch as it was presented to the Hygienic Congress at Buda Pesth recently, and as presented in the last number of the *American Journal of Medical Sciences*. The other question which has been hinted at, Is tracheotomy ever beneficial or justifiable? I cannot go into my record as far as tracheotomy is concerned, but I will briefly say that in the present year I have operated eight times for diphtheritic laryngitis where there was foreign infection, where the cases never were isolated and where the cases sprang up; in one instance seven cases surrounded the one I operated upon. In one of those cases we had to feed the patient by the rectum. Four of those patients got well.

Dr. F. J. Thornbury: I think the only treatment now is the anti-toxine treatment, and this fact has been established in practice and by animal experimentation.

Dr. Young: Mr. President, I wish only to state in regard to my paper, that the real object was to draw out the idea that the bacilli are developed in the blood from spores, and may compose the so-called toxines which are present, but the composition of which is unknown; and that these semi-developed germs nevertheless exist, though they have not been identified by the microscope.

---

Dr. E. W. Kellogg strongly advises frequent *large* doses of turpentine in diphtheria, which should be persisted in as long as there is any chance whatever. He reports twenty-six cases, all involving the larynx, of which fourteen cases, or fifty-four per cent., recovered.—*Med. Mirror.*

